# Child-related factors associated with depressive symptoms among mothers of school-going children in urban Bangladesh: A cross-sectional study

**DOI:** 10.1371/journal.pone.0304480

**Published:** 2024-05-29

**Authors:** Sharmin Sultana, Faisal Muhammad, A. B. M. Alauddin Chowdhury, Sabuj Kanti Mistry

**Affiliations:** 1 Department of Public Health, Daffodil International University, Daffodil Smart City, Birulia, Savar, Dhaka, Bangladesh; 2 Department of Public Health, Miltonbridge University, Mogadishu, Somalia; 3 Department of Public and Community Health, Faculty of Medicine and Health Sciences, Frontier University Garowe (FUG), Puntland, Somalia; 4 Otu Institute of Research and Training (OIRT), Kano, Nigeria; 5 ARCED Foundation, Mirpur, Dhaka, Bangladesh; 6 School of Population Health, University of New South Wales, Sydney, NSW, Australia; Uttara Adhunik Medical College, BANGLADESH

## Abstract

**Objective:**

This study aimed to identify the child-related factors associated with depressive symptoms among mothers of school-going children in Dhaka city of Bangladesh.

**Methods:**

The study followed a cross-sectional design and was conducted between June and December 2019 among mothers of school-going children from Dhaka City, Bangladesh. A multistage sampling technique was adopted, and a total of 324 mothers of school-going children studying in the same school for at least six months were selected. Depressive symptoms of mothers were measured using a 20-item Self-Rating Depression Scale weighted to 100 percent, with 25–49 categorized as no depression and ≥ 50 as having depression. A binary logistic regression model was executed to identify the child-related factors associated with depressive symptoms among mothers. All statistical analyses were performed using the statistical software, Stata (Version 14.0).

**Results:**

More than half of the participants (54.3%) were aged 40 years or above and had up to HSC level education (52.5%). The majority of the participants were homemakers (67.0%), mothers of a girl child (53.1%), and had a family income of 50,000 BDT or more (52.8%). Adjusted analyses revealed that the mother’s depressive symptoms were associated with their child’s frequent complaints of headaches or stomach aches (aOR = 13.19, 95% CI 3.03–57.37), having an injury (aOR = 4.05, 95% CI 1.44–11.41), and unfriendly relationship with mothers (aOR = 21.46, 95% CI 5.04–91.28).

**Conclusion:**

The present study highlighted several child-related factors that are associated with depressive symptoms among mothers that need to be considered while designing any intervention to address depressive symptoms among mothers of school-going children. It is also important to counsel mothers and fathers of the children about the importance of having a sound mother-child relationship while addressing depressive symptoms among mothers.

## Introduction

Depressive symptoms are prevalent, usually occurs in episodes [1], and often can become persistent and chronic [2]. They stand as a significant source of disability globally, with a particular impact on women [3]. According to the World Health Organization, 6% of women experience depression, which is 4% among men [4]. Women are especially vulnerable to depressive symptoms after the birth of a child, especially in the first year of their child [5]. It is estimated that more than half of mothers of young children may experience depressive symptoms at some point in their lives [6]. Sadness, fatigue, negative attitude, lack of interest, difficulty in thinking clearly, as well as withdrawal and intrusiveness are major noted symptoms of depression among mothers [7].

If mothers are suffering from depressive symptoms it can be challenging to provide consistent, attentive, and responsive childcare for them, and can disrupt effective parenting [8]. Depression in mothers often results in negative parenting (less warmth), lack of care to their child resulting to frequent access to acute health care services, insecure attachment, as well as aggressive behaviour and cognitive vulnerabilities to depression amongst their children [9]. Research indicates that when mothers experience depressive symptoms, it can significantly impact their children’s mental health outcomes [9, 10]. Maternal depressive symptoms may contribute to a range of adverse effects on children, including increased vulnerability to emotional and behavioral difficulties, higher risk of developing depressive or anxiety-related issues, lower intellectual development and potential challenges in their overall psychological well-being [9, 11]. Postpartum maternal depressive symptoms and those in later stages have also been identified as risk factors for impaired development among children [12].

Research suggests that strong mother-child attachment significantly influences various aspects of a child’s future well-being, encompassing physical, cognitive, and psychosocial outcomes in adulthood [13]. A positive and secure attachment fosters healthy connections and interactions for the child, potentially impacting their future parenting experiences [14]. Researchers pointed out that the mother-children relationship begins during pregnancy and continues throughout the child’s life [15]. For instance, it is evident that a better mother-children relationship during pregnancy is associated with a better maternal-infant relationship after birth [16]. Poor mother-child relationship can lead to long-term adverse effects on a child’s development and mother-infant relationships [9, 12].

Although approximately 90.0% of the world’s children and adolescents reside in low- and middle-income countries [17], there is still a dearth of evidence around the depressive symptoms among the mothers of these children. Some prior studies conducted overseas documented the link between adverse child health outcomes and negative mother-child relationships with maternal depressive symptoms [18, 19]. While there is very limited evidence available from Bangladesh on depression among mothers, a recent study carried out in Bangladesh documented that 20.1% of the mothers had severe depressive symptoms [7]. This study also highlighted the interconnectedness between children’s lifestyles and certain behavioral factors, such as the child’s temperament, extensive television viewing, and internet usage, and the increased prevalence of depression among mothers [7]. Notably, while not explicitly labeled as unhealthy, these attributes of a child’s life have shown correlations with elevated levels of maternal depression, suggesting a potential area of influence that warrants further exploration. Understanding child-related factors associated with depressive symptoms among mothers holds significance due to their potential impact on the well-being of both mothers and children. These factors can play a crucial role in shaping mothers’ mental health, and influencing parenting behaviors. Examination of these associations would enable to uncovering the potential pathways through which the child’s characteristics or behaviors may contribute to or mitigate mothers’ depressive symptoms, thereby informing targeted interventions that promote healthier family environments and mothers’ mental well-being.

While there is global evidence that maternal depressive symptoms and mother-child bonding are associated with development and well-being of the children, limited research has focused on identifying the specific child-related factors contributing to these symptoms in the context of Bangladesh. The present study fills this gap in the literature and specifically seeks to investigate whether specific factors such as a school-going child’s academic performance, health status, and behavioural characteristics are associated with depressive symptoms in their mothers.

## Materials and methods

### Study design, setting and participants

This cross-sectional study was conducted during June and December 2019 in 12 randomely selected schools from two city corporations of Dhaka City, Bangladesh, among mothers of school-going children. The study population included all mothers of children who were students at a school (class V-VIII) in Dhaka city.

### Required sample size

A sample size of 324 mothers was determined considering 18.0% prevalence [20] with a 4.18% margin of error, at the 95% level of confidence. Sample size was calculated using the following formula:

$$n=(Z^{2}*p*q)/E^{2}$$

Where, n = sample size; Z = z-score (for 95% confidence interval which is 1.96; p = estimated percentage of population as expected (18.0%); q = 100-p; E = margin of error (4.18%).

Therefore, sample size (n) = (1.96^2^ * 18 * 82) / (4.18)^2^ = 324.

### Questioannire development

The research team members crafted the questionnaire in English and then translated it into Bengali language. In the next step, Bengali questionnaire was back-translated in to English to ensure the contents’ consistency. The initial version of the Bengali questionnaire was piloted with some mothers of school-going children (n = 17) who were not included in the study to refine the language of the questionnaire. We did not receive any corrections or modifications to the piloted questionnaire.

### Sampling and data collection

**Fig 1:**
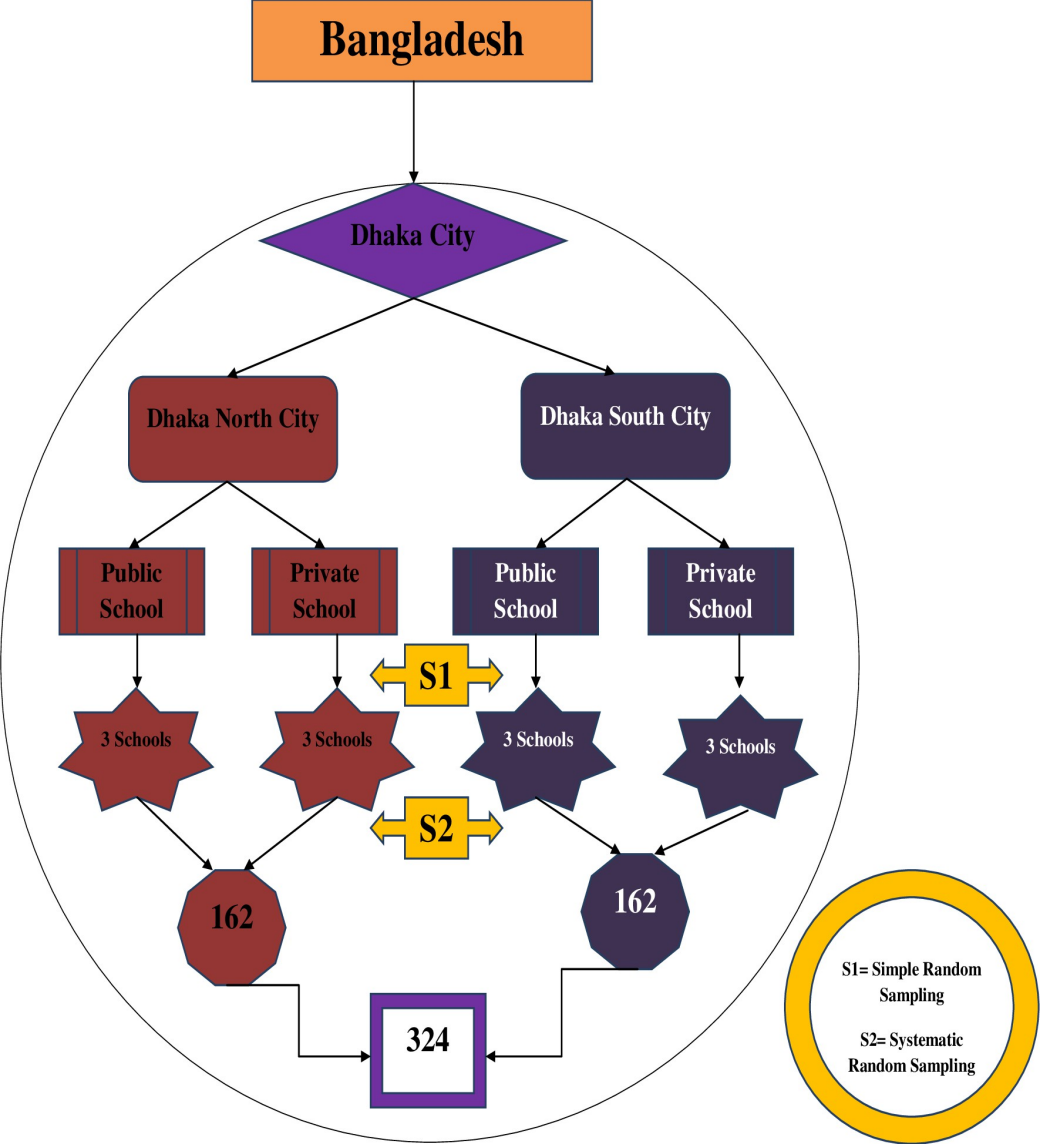
A multistage sampling technique.

A multistage random sampling technique (Fig 1) was followed. Initially, the list of all public and private schools from both of the city corporations of Dhaka (Dhaka North and South) was collected from the website of the Board of the Intermediate and Secondary Education, Dhaka (https://dhakaeducationboard.gov.bd/index.php/site/subdomain). We categorized the schools as private or public and in terms of North or South City corporations. In the next phase, we randomly selected three public and three private schools from each of the two City corporations. Subsequently, the list of all students (class V-VIII) was collected from the registers of the selected schools. The list of students served as the sampling frame for the study and using computer-generated random numbers, mothers of these students were randomly recruited to achieve the required sample size. The detailed sampling strategy is presented in Fig 1. Mothers whose children had attended the same school for six months met the inclusion criteria.We also excluded the mothers who were not willing to participate.

The data was collected from the sampled population using the pre-tested semi-structionred questionnaire through face-to-face interviews conducted by the first author (SS) and each interview took around 30–45 minutes.

### Study variables and measurements

#### Outcome variable

Depressive symptoms were the primary outcome variable, measured using the 20-item Zung Self-Rating Depression Scale (SDS) [21, 22]. The SDS has been previously used for measuring depressive symptoms in the adult population in Bangladesh [23]. The scale comprises of ten positively worded and ten negatively worded questions. Each question is assigned a score ranging between 1 and 4 (a little of the time; some of the time; a good part of the time; most of the time). This is weighted to 100% and classified into four categories: no depression (25–49), mild depression (50–59), moderate depression (60–69), and severe depression (≥70) [21]. We found the scale reliable with a Cronbach’s alpha of 0.89 among our study participants.

#### Explanatory variables

Explanatory variables which were determined based on review of previous literature [6, 7, 24] considered in this study were age, religion, educational qualification, occupation, child’s gender, monthly family income (BDT), family type, child’s sickness, chronic disease, injury, friendly relationship with mother, overall relationship with mother, school attendance and academic performance.

### Bias

We envisaged there are potentials for selection and information bias in the study [25, 26]. We tried control for any potential bias that may introduced in the study process. For example, we adopted a random sampling technique to avoid any potential selection bias. The interviews were conducted by the first author (SS) only which diminishes the potential inter-interviewer bias. However, the potential for recall bias and presence of any unobserved confounder should be considered with caution.

### Data analysis

Descriptive analyses were performed to explore the distribution of the variables. The Chi-square test was performed to compare differences in the prevalence of depressive symptoms by explanatory variables, with a 5% level of significance. To explore the child-related factors associated with depressive symptoms, a binary logistic regression model was executed. We only included those variables in the regression model which had a P-value of <0.25 in the bivariate analysis. We reported the crude odds ratio (COR), the adjusted odds ratio (AOR), and the associated 95% confidence interval (CI). The statistical software, Stata (Version 14.0) was used for all analyses.

### Ethical issue

Ethical approval was sought from the Research Ethics Committee, Faculty of Allied Health Sciences (REC-FAHS), DIU (Ref: FAHSREC/DIU/2021/1006-31). All procedures performed in the study involving human participants were in accordance with the ethical standards of the institutional and/or national research committee and with the 1964 Helsinki Declaration and its later amendments or comparable ethical standards. Both written and verbal consents were taken from each participant before the interview. Anonymity and confidentiality were strictly maintained.

### Patient and public involvement

Patients and/or the public were not involved in the development of research questions, study design, conducting study, and result dissemination.

## Results

### Socio-demographic characteristics of the participants

The majority of the participants were aged ≥ 40 years (54.3%), Muslims (85.5%), had HSC-level education (52.5%), homemakers (67.0%), and mothers of a girl child (53.1%). More than half of the participants (52.8%) had a family income of 50,000 BDT or more, and most of them (82.1%) lived in a nuclear family (Table 1).

**Table 1:** Socio-demographic status of the respondents (n = 324).

Characteristics	n	%
Age (year)		
< 40	148	45.7
≥ 40	176	54.3
Religion		
Muslim	277	85.5
Non-Muslim	47	14.5
Educational Qualification		
Up to HSC	170	52.5
Above HSC	154	47.5
Occupation		
Working at home (Home maker)	217	67.0
Working outside (Job, business)	107	33.0
Gender of child		
Boy	152	46.9
Girl	172	53.1
Monthly family income (BDT)		
< 50,000	153	47.2
≥ 50,000	171	52.8
Types of family		
Nuclear	266	82.1
Joint	58	17.9

### Child-related factors and depressive symptoms among mothers

Around one in ten mothers (13.0%) reported that their children frequently complained of sickness. A few mothers reported that their children had a chronic disease (5.3%) and injury (12.4%). The majority of the mothers said that they have a friendly relationship (84.0%) and a good relationship (86.7%) with their children. Likewise, most of the mothers mentioned that their children were regular in their class (84.6%), and got satisfactory results in their last examination (51.9%) (Table 2).

**Table 2:** Bivariate analysis of child’s factors and mothers’ depressive symptoms (n = 324).

Characteristics	Overall n (%)	%Depressive symptoms
Overall	324(100.0)	57.7
Child often complains of headaches, stomach-aches, or sickness*		
Yes	42(13.0)	95.2
No	282(87.0)	52.1
Chronic disease of the child*		
Yes	17(5.3)	100.0
No	307(94.8)	55.4
Child face any accident*		
Yes	40(12.4)	87.5
No	284(87.7)	53.5
Friendly relationship with the child*		
Yes	272(84.0)	64.6
No	52(16.1)	57.9
Rating of the relationship with the child*		
Good	281(86.7)	51.3
Neither good nor bad	39(12.0)	100.0
Bad	4(1.2)	100.0
Child’s school attendance*		
Regular	274(84.6)	50.0
Irregular	50(15.4)	100.0
Child’s academic performance in the last examination*		
Unsatisfactory	120(37.0)	39.2
Satisfactory	168(51.9)	61.9
Pass	25(7.7)	100.0
Fail	11(3.4)	100.0

* = *p value <0.010*

### Factors associated with depressive symptoms among mothers

In the unadjusted analysis, it was found that mothers whose children often complained of sickness were 18 times more likely to have depressive symptoms (cOR = 18.37, 95% CI: 4.36–77.46) than those whose children did not often complain. Likewise, mothers of children who had an injury were 4 times more likely to have depressive symptoms (cOR = 6.08, 95% CI: 2.31–15.97) and mothers who had an unfriendly mother-child relationship were more than 24 times more likely to have depressive symptoms (cOR = 24.64, 95% CI:5.88–103.27) compared to their counterparts (Table 3).

**Table 3:** Child-related factors associated with depressive symptoms among mothers (n = 324).

Characteristics	cOR^1^	95% CI	P	aOR^2^	95% CI	P
The child often complains of sickness (headaches, stomach-aches)						
Yes	18.37	4.36, 77.46	<0.001	13.19	3.03, 57.37	0.001
No	Ref			Ref		
Child injury						
Yes	6.08	2.31, 15.97	<0.001	4.05	1.44, 11.41	0.008
No	Ref			Ref		
Child ‐ mother-friendly relationship						
No	24.64	5.88, 103.27	<0.001	21.46	5.04, 91.28	<0.001
Yes	Ref			Ref		

^1^Crude odds ration; ^2^Adjusted odds ration; some factors were excluded from the regression model due to collinearity; The model was adjusted with mothers’ age, religion, educational qualification, occupation, gender of the child, monthly family income (BDT), and family type

Meanwhile, the adjusted analysis revealed that mothers whose children often complain of sickness had more than 13 times higher odds of having depressive symptoms (aOR = 13.19, 95% CI: 3.03–57.37) compared to those whose children did not often complain of sickness. Similarly, mothers of children with injury had more than 4 times higher odds of depressive symptoms (aOR = 4.05, 95% CI: 1.44–11.41) and mothers who had an unfriendly mother-child relationship had more than 20 times higher odds of depressive symptoms (aOR = 21.46, 95% CI:5.04–91.28) compared to their counterparts (Table 3).

## Discussion

The present study investigated the child-related factors associated with depressive symptoms among mothers of school-going children from Dhaka City, Bangladesh. We found that mother’s depressive symptoms were associated with several child-related factors, such as frequent complaints of headaches or stomachaches, injury, and an unfriendly relationship with mothers.

In our study, we observed depressive symptoms among 57.7% of mothers, a figure in line with findings from a similar study in Bangladesh that reported a prevalence of 52.0% [24]. While there are limited studies conducted in Bangladesh exploring depressive symptoms among mothers of young children, a wide variation in prevalence of maternal depression was reported in studies conducted in Taiwan (27.7%) [27], USA (28.0%) [28], and Honduras (69.6%) [29].

Our study identified a correlation between a child’s illness and mothers’ depressive symptoms. Previous studies conducted in the similar settings also demonstrated a correlation between maternal depression and child’s poor health and nutritional outcomes [30, 31]. Additionally, other research has highlighted that parents of chronically ill children experience heightened anxiety and depression [32]. The concern and anxiety of mothers regarding a child’s illness extend beyond immediate care to encompass worries about the child’s recovery, ensuring optimal treatment [33], and re-establishing the child’s educational and social routines [34].

Previous research highlighted the profound impact of child injuries, particularly road traffic injuries, on maternal stress [35]. Our investigation delved into how mothers of children who experienced injuries, including road accidents, grappled with fear and anxiety, often resulting in depressive symptoms. While our focus was not exclusively on road traffic injuries, the wider context of child injuries, including those occurring on roads, was a significant factor in understanding the link between a child’s injury and the subsequent impact on maternal mental well-being. According to our study, mothers who experienced a child’s injury had higher odds of experiencing depressive symptoms. Our results lined up with a population-based birth cohort study that found a link between child injuries and maternal depression [36]. A nationally representative survey conducted in the USA also reported a correlation between maternal depressive symptoms and the risk of child injury [37]. Moreover, according to a population-based cohort study done in Denmark, a mother’s depression symptoms were associated with the child’s injuries, particularly during the first year of life.[38].

The present study also reported that lack of a good mother-children relationship was associated with increased depressive symptoms among mothers of school-going children. Previous research also found that children who have negative relationships with their mothers are more likely to have psychosocial and behavioral problems [39, 40]. A recently published study also documented that maternal mental health is related to maternal-infant relationships [41].

### Implications for policy and practice

In 2018, a new Mental Health Act was passed by the Parliament of Bangladesh.. Additionally, in 2019, the Ministry of Health approved a new Mental Health Policy that emphasizes community-based services and support for individuals living with mental illnesses, decentralization, and a shift from a medical to a psychosocial treatment approach [42]. The results of this study also have important policy implications given this recent policy inclusion. Child-related health outcomes and mother-children relationships must be considered important in the policy discourse when the management of depressive symptoms among mothers is concerned. Policymakers need to consider providing mental health support for mothers of school-going children with sickness, who can be flagged by school authorities.

Additionally, counseling is also recommended for mothers as well as fathers of school-going children to ensure a friendly relationship with their children. In particular, fathers should allocate more time for their children and their partner/s which will allow them to have a better mother-child relationship. From a policy standpoint, it could be imperative to launch awareness-raising campaigns to improve mothers’ and other family members’ understanding of issues related to mental health, ensure timely identification of mental health symptoms, support treatment, and medication adherence, and open discussions about depression to eliminate stigma. Family physicians could play a pivotal role in this regard. In this approach, it needs to be cautiously maintained that mothers are not blamed for their aggravating depressive symptoms. Also, it needs to be ensured that mothers are not labeled as depressed which can result in widespread stigmatization of them. Finally, aligned with the findings of the research it is important to consider improving the mother-child relationship to meet the Sustainable Development Goal (2015–2030) of reducing mental health problems in Bangladesh [43].

### Limitations of the study

This is the first study that explored the child-related factors associated with depressive symptoms among mothers to the best of our knowledge. Nevertheless, there are certain limitations of the study. First, because this is a cross-sectional study, we are unable to establish a causal relationship between the mother’s depressive symptoms and the child-related factors. Also, we interviewed study participants and therefore, can be subjected to recall and reporting bias. Moreover, the study findings cannot be generalized to Bangladesh as the participants were selected only from Dhaka city. Furthermore, it can be a concern among the participants with limited educational achievements how they understood and interpreted the depressive symptoms when they were interviewed. Finally, we would like to acknowledge that due to several administrative issues associated with the selection of the schools and concomitant data collection, it took around six months for the data collection. However, we could not control any potential seasonal variations in depressive symptoms among mothers.

## Conclusions and recommendations

Our study findings reveal that more than half of the mothers exhibited symptoms of depression, posing a serious concern. This investigation underscores a notable link between a mother’s depressive symptoms and various factors such as her child’s illness, injuries, and strained relationships. Mitigating a mother’s depression necessitates not only the support of her spouse and social network but also heightened parental awareness of early childhood behavioral cues. Counseling mothers on fostering positive relationships with their children emerges as a crucial strategy in addressing depressive symptoms. Additionally, policymakers and public health professionals should consider child-related factors when formulating interventions aimed at managing depressive symptoms among mothers with school-aged children.

## Supporting information

S1 File (DTA)
